# Association between societal costs and treatment response in children and adolescents with ADHD and their parents. A cross-sectional study in the Netherlands

**DOI:** 10.1186/s40064-015-0978-7

**Published:** 2015-05-15

**Authors:** Annemarie van der Kolk, Clazien AM Bouwmans, Saskia J Schawo, Jan K Buitelaar, Michel van Agthoven, Leona Hakkaart-van Roijen

**Affiliations:** Radboud University Medical Centre, Donders Institute for Brain, Cognition and Behavior, Department of Cognitive Neuroscience, P.O. Box 9010, 6500 GL Nijmegen, the Netherlands; Janssen-Cilag BV, P.O. Box 90240, 5000 LT Tilburg, the Netherlands; Institute for Medical Technology Assessment (iMTA), P.O. Box 1738, 3000, DR Rotterdam, the Netherlands; Karakter Child and Adolescent Psychiatry University Centre, Reinier Postlaan 12, 6525 GC Nijmegen, the Netherlands; Gilead Sciences B.V., Amsterdam, The Netherlands

**Keywords:** ADHD, Societal costs, Burden of illness, Treatment response, Cross-sectional

## Abstract

Attention Deficit Hyperactivity Disorder (ADHD) is associated with considerable burden of illness at a patient, family and societal level. Although pharmacological treatment is recommended by authoritative guidelines, evidence on its influence on the broader burden of illness is limited. As treatment induces costs, proper healthcare decision making requires evidence on the associated societal costs or benefits and particularly the difference that response to treatment can make.

Data on ADHD related resource use of patients 8–18 years and parents were collected by means of a cross-sectional, online survey amongst members of the Dutch parent association. Children were stratified to responders and non-responders to treatment according to pre-defined expert definitions.

Analyses were performed on 618 questionnaires (428 responders; 190 non-responders to treatment). Children were 11.8 years on average and mainly boys (82 %). Total monthly costs for children were €578 and €839 for responders and non-responders, respectively (p = 0.021), with a breakdown to direct medical costs (€322 vs. €512; p = 0.068), direct non-medical costs (€222 vs. €296; p = 0.090), and indirect non-medical costs (€34 vs. €57; p < 0.001). For parents, total costs were €246 vs. €399 for the responding and non-responding children, respectively (p = 0.006), with a breakdown to direct medical costs (€130 vs. €211; p = 0.010) and indirect non-medical costs (€116 vs. €181; p = 0.092). Total monthly costs of children and their parents together were €824 and €1228 for responders and non-responders to treatment, respectively (p = 0.002).

These results stress the importance of a focus on response to treatment, not only beneficial for patients and their family, but also resulting in considerable societal benefits.

## Introduction

Attention Deficit Hyperactivity Disorder (ADHD) is a heterogeneous psychiatric disorder characterized by a chronic pattern of age-inappropriate inattention, hyperactivity and impulsivity with a worldwide-pooled prevalence of 5.3 % (Asherson 2004; Polanczyk et al. 2007; Swanson et al. 2009). Adverse consequences of ADHD may be far-reaching and symptoms often persist into adulthood (Barkley 2008; Coghill et al. 2008; Faraone et al. 2006; Michielsen et al. 2012).

ADHD is associated with a considerable burden of illness at both the patient, family, and societal level (Birnbaum et al. 2005; Doshi et al. 2012; Erskine et al. 2014; Le et al. 2013). Several studies concluded that ADHD has a negative impact on family, school and environmental level reflected in a lower quality of life (QoL), increased use of healthcare resources and risk for injuries, substance abuse, delinquency, and driving violations (Danckaerts et al. 2009; De Ridder & De Graeve 2006; Hakkaart-van Roijen et al. 2007; Mannuzza et al. 2008; Thompson et al. 2007; van den Ban et al. 2014; van der Kolk et al. 2014). The influence of ADHD on others than the patient is previously shown by productivity losses of parents and an increased use of healthcare resources by family members (Birnbaum et al. 2005; Swensen et al. 2003). Treatment by medication has been shown to decrease ADHD symptoms and to consequently improve the level of functioning. Medication is recommended by authoritative treatment guidelines worldwide as a first-line treatment option in case of ADHD (Banaschewski et al. 2006; Canadian Attention Deficit Hyperactivity Disorder Resource Alliance 2010; National Institute for Health and Clinical Excellence 2006; Trimbos-instituut et al. 2005; Wolraich et al. 2011). A recent systematic review of long-term outcomes of ADHD treatment and non-treatment supports the premise that without accurate treatment, people with ADHD experience poorer long-term outcomes (Shaw et al. 2012). Also, non-adherence to treatment could jeopardize treatment outcome (van den Ban et al. 2010). However, evidence is limited regarding the influence of treatment by medication on health care resource use of ADHD patients and their families, and the associated economic burden. This is a hindrance for the evaluation of the cost-effectiveness of current and future pharmacological interventions for ADHD (Le et al. 2013).

Studies on health care resource use and costs are preferably conducted from the societal point of view (Jonsson 2009). A profound societal analysis includes medical (inside the health care sector) and non-medical costs (outside the health care sector), both direct (e.g. a visit to a doctor) and indirect costs (e.g. absence from work due to illness) (Jonsson 2009). This broad societal perspective is especially indicated in evaluating the economic burden of chronic diseases like Alzheimer’s disease, Multiple Sclerosis, Rheumatoid Arthritis and ADHD. These are all examples of disease areas where new innovations could lead to reductions in costs for society due to reductions in productivity gains and reductions in costs for community and informal care (Jonsson 2009). However, only few studies have monetized the impact of ADHD systematically by providing an impression of the overall impact of ADHD from a societal perspective with a comprehensive measurement of indirect costs (Doshi et al. 2012; Le et al. 2013). A recent review concluded that data is lacking to draw conclusions regarding the relative cost-effectiveness of different pharmacological agents in ADHD (Le et al. 2013). In recent years, the issue of non-compliance with (pharmacological) treatment is studied further to evaluate the clinical and economic effects of the management of compliance in patients (Hiligsmann et al. 2012). In ADHD treatment, persistence to treatment is frequently suboptimal indicating impaired compliance (van den Ban et al. 2010). Poor compliance, or in broader terms poor response to treatment, reduces the potential benefits from therapy. To our knowledge, costs in children with ADHD have not been studied in association to response to treatment.

This paper therefore presents a cost study on ADHD from the societal perspective including costs related to resource use and productivity losses. As medication is recommended by guidelines as a first-line treatment option and response to treatment is associated with potential treatment benefit, proper healthcare decision making requires evidence on the associated societal costs or benefits and particularly the difference response to treatment can make.

### Methodology

The present study was part of a larger study on the burden of ADHD from a societal perspective including QoL (van der Kolk et al. 2014). The current study analyses all direct and non-medical indirect costs related to ADHD (Table 1). Data on healthcare consumption of children and their parents and productivity losses of parents were collected by means of a cross-sectional, online survey that was completed by parents.

**Table 1:** Types of resource use included for children (Ch^a^) and parents (P^b^) or both groups (Ch-P)

	Direct	Indirect
Medical	Medical consultations (Ch-P)^c^	^d^
	Skills training (Ch)	
	Parent training (P)	
	Day-care treatment (Ch)	
	Hospitalization (Ch)	
	Medication (Ch-P)	
Non-medical	Remedial teaching at school (Ch)	Agency for child welfare (Ch)
	Weekend care (Ch)	Police (Ch)
	Care after school (Ch)	Special education (Ch)
	Diet (Ch)	Productivity losses
		Absenteeism (P)
		Presenteeism (P)

^a^Ch: Item included in the cost calculation of the children only

^b^P: Item included in the costs for parents referred to their own health consumption because of their child’s ADHD

^c^Ch&P: Item included in both the cost calculation for the children as well as in the cost calculation for the parents, but costs for children are not included in the costs for parents

^d^Indirect medical costs are excluded according to the Dutch guidelines for pharma-economic research (College voor zorgverzekeringen afdeling pakket 2006)

### Study sample

The study sample was derived out of the members of the Dutch association *Balans* for parents of children with developmental disorders (amongst others, but not limited to ADHD, Asperger’s syndrome, Dyslexia, oppositional defiant disorder (ODD), and conduct disorder (CD)). Parents of children with ADHD were asked via the magazine of the association to join the study online by means of a personal login code. Inclusion criterion: being a parent of a child aged 8–18 years diagnosed with ADHD. Because of this membership, it was assumed valid to rely on the parents’ knowledge about the ADHD diagnosis of their child. Due to anonymity it was not possible to verify whether the diagnosis was indeed valid.

The questionnaire was available from 01-09-2010 until 03-10-2010. The parent taking care of the child most of the day was asked to complete the questionnaire. Parents were not reimbursed for their participation in any way. In case the parent had more than one child with ADHD, parents were asked to answer the questions for their youngest child. Since the study did not interfere with clinical practice, according to Dutch regulation no approval of an ethics committee was needed. Also, with the methodology of this study focusing on parents instead of patients, it was not obligatory to use informed consent forms. Nevertheless, the protocol took into account all privacy aspects and all necessary efforts were taken in this study to protect privacy. Participants were informed on the purpose of data collection (scientific publication), respondents were fully free to decide to join and were able to exit the questionnaire at any time. Due to the login code that was distributed by Balans magazine, only members of Balans were able to access the questionnaire. The questionnaire could be completed only once per IP address, but these IP addresses were not saved and/or traceable so participants’ anonymity was guaranteed. Also, adequate security measures were employed in order to prevent unauthorized access, manipulation to or disclosure of the personal data although these were already unidentifiable when stored.

### Responder groups

In order to investigate the association between costs and response to treatment, descriptions for response to treatment had to be defined since these were not readily available. These descriptions were created by five Dutch ADHD experts. This number of five experts was considered appropriate for the study, in accordance with minimum requirements of a Delphi panel (Evans & Crawford 2000). The panel of experts was selected based on their experience in the field of ADHD treatment, and a scientific focus. The experts included were child-and youth psychiatrists (n = 5), both male (n = 4) and female (n = 1) and aged 43 to 55 (mean 49.8). The experts had 10 to 22 years of experience with ADHD medication and treated 30–90 ADHD patients per month. The experts were consulted independently and were not aware of the identity of the other experts joining the panel. An expert not joining the Delphi panel verified the questions proposed to the experts in advance. The questions for the panel were sent and returned by email. After all experts had returned the questionnaires, their answers were combined. The proposals for the final answers, as well as the anonymous individual answers of the participants were reported to the experts after round 1. In the second round, experts were asked whether they liked to change their previous answers, based on the proposal for the final answer. Before distributing the questions to the experts, it was decided that consensus was supposed to be reached after two rounds of answers when (a) feedback of the experts was clear and (b) when experts did not all change their answers based on the mean of the feedback of the first round. The experts indicated that two assumptions were crucial for the foundation of the definitions for response to treatment and non-response to treatment. First, based on an earlier study, response to medication had to be defined by recovery both at home and at school in order to reflect a proper description of the child’s daily functioning (functioning - F) (Buitelaar et al. 1995; Hodgkins et al. 2013). Second, as compliance to medication is known to play an important role in the child’s functioning, it was considered necessary to include a statement on the child’s compliance as well (compliance - C) (Charach & Fernandez 2013). Theoretically, this led to the following combinations:

- (C^+^; F^+^): Compliance to prescribed daily dose of medication, no problems in functioning.
- (C^−^; F^−^): Non-compliance to prescribed daily dose of medication, problems in functioning.
- (C^−^; F^+^): Non-compliance to prescribed daily dose of medication, no problems in functioning.
- (C^+^; F^−^): Compliance to prescribed daily dose of medication, problems in functioning.

The experts decided to exclude combinations (C^−^; F^+^) and (C^+^; F^−^). Experts considered (C^−^; F^+^) to describe children not requiring medication, as no problems arise when medication is not taken according to prescription. (C^+^; F^−^) was considered to reflect several situations in which proper compliance is not translated into proper functioning: inappropriate dosing, inappropriate medication, or presence of an undiagnosed but relevant comorbid condition. The design of the study precluded to analyze these situations. The two remaining combinations, (C^+^; F^+^) and (C^−^; F^−^), were the basis for responder group definition. As it was deemed necessary to provide parents with a hands-on description of these groups, the experts operationalized the combinations to the following descriptions based on treatment compliance and functioning in family, school and the environmental level:

- Responder to treatment: *‘Your child uses the prescribed daily dose of medication. Using this treatment, your child’s functioning is fine and there are no noteworthy problems at home, at school, with peers or during leisure time’.*
- Non-responder to treatment: ‘*The daily dose of medication used by your child differs from the dose prescribed. Using this treatment, your child’s functioning is quite fine, but he/she is hampered by problems at home, at school, with peers or during leisure time for short periods of time’.*

### The questionnaire

The questionnaire on healthcare consumption and productivity losses for patients with psychiatric disorders (TiC-P) was used, which is designed for self-report and is focused on psychiatric disorders like ADHD (van Roijen et al. 1996). Two versions are available, one for adults and one for adults to describe their children (Bouwmans et al. 2013b; Bouwmans & Hakkaart-van Roijen 2013b). The selection of this questionnaire was based on feasibility, reliability and validity combined with comprehensiveness of cost items and ability of online use (Bouwmans et al. 2013a). The questionnaire had been used on an ADHD sample in another study before (Hakkaart-van Roijen et al. 2007). In the present study, both TiC-P versions were used. The questionnaire was adjusted using information from a previously performed study focused on societal cost related to ADHD as is allowed by the TiC-P manual (Bouwmans & Hakkaart-van Roijen 2013a; Hakkaart-van Roijen et al. 2007). The recall period was one month for both medical consumption and productivity losses. TiC-P starts with general demographic questions on gender, age, (type of) education, marital status, and job.

TiC-P questionnaires are generic, meaning that items are not related to a specific disorder. In this study, resource use was supposed to be related to the ‘target disorder’ ADHD. Parents were provided with examples of costs that were either related (visiting a doctor for ADHD problems) or non-related to ADHD (visiting an ear, nose and throat specialist for ear tubes) to aid them in making a correct distinction. This could also mean costs for parents getting coaching and/or parenting tips; this is only necessary because of their child with ADHD and therefore these costs are considered related to ADHD. Costs for parents referred to their own health consumption only plus costs due to productivity losses.

Table 1 presents the cost items that were included, Table 2 presents the unit costs per cost item. For children, additional questions to Table 2 were focused on costs for diet, the use of ADHD medication, use of special education and absence from school or sports. Costs of productivity losses were focused on productivity losses of paid work including absenteeism (absence from work) and presenteeism (reduced productivity while at work). Non-paid work was not included in this study, since parents of children aged 8–18 were expected to have limited voluntary activities.

**Table 2:** Unit costs used for children (Ch) and parents (P) or both groups (Ch-P) in Euro 2012

	Ch-P	Unit costs (Hakkaart-van Roijen et al. 2011)^a^
Consultation per session	-	-
General Practitioner	Ch-P	29.73
Psychologist^b^	Ch-P	84.95
Social worker	Ch-P	69.02
Psychotherapist, first line^c^	Ch-P	81.76
RIAGG^d^	Ch-P	81.76
Phychiatrist outpatients’ department	P	109.37
Medical specialist	Ch	109.37
Emergency Room	Ch	160.34
Physiotherapist	Ch	38.23
Occupational therapist	Ch	23.36
Speech therapist	Ch	35.04
Alternative therapist	Ch-P	50.00 (Nederlandse Orde van Alternatieve Genezers 2013)
Paramedical (physiotherapist, occupational and alternative therapist)	P	37.12
School doctor	Ch	29.73
Company doctor	P	29.73
Trainings per session	-	-
Social skills training/creative therapy/self regulation^e^ - individual	Ch	181.58
Social skills training/creative therapy/self regulation^e^ - group	Ch	100.00
Psychological education^f^/parent training/family coach - individual	P	181.58
Psychological education^f^/parent training/family coach - group	P	100.00
Day-care treatment	Ch	173.62
Hospitalization	Ch	246.35
Agency for child welfare	Ch	69.02
Weekend care (hours)	Ch	13.27
Police (contacts)	Ch	69.02
Remedial teaching at school (hours)^g^	Ch	55.00 (Landelijke beroepsvereniging remedial teachers 2014)
Care after-school (hours)	Ch	5.93 (Nibud 2013)

^a^Dutch cost manual unless specified otherwise and prices were indexed to 2012 prices

^b^Second line care aimed at more severe complaints needing a longer treatment duration

^c^Short duration (up to 8 sessions) aimed at mild to moderate complaints

^d^Regional institution for ambulant psychiatric health care

^e^To control or direct oneself according to rule

^f^Provide information to parents regarding coping with the ADHD of their child

^g^Special teaching for backward and slow learners

### Cost calculation

Bottom-up methodology was used to calculate total direct medical costs; that is, the total number of medical contacts was multiplied by the reference prices based on the Dutch manual for cost studies (prices were updated to 2012 prices) (Hakkaart-van Roijen et al. 2011). A visit to ‘RIAGG’ (Dutch mental health facility: a combination of 1^st^ and 2^nd^ line care) was assumed to have similar costs as 1^st^ line mental health care in case of ADHD. Unit costs for a visit to a school doctor and a company doctor were assumed to be similar to a general practitioner visit as costs for a school doctor and a company doctor were not defined in the costing manual. These assumptions were confirmed by a specialist in costing studies (LH). Costs for individual and group trainings were not specified in the costing manual. For individual sessions, costs for ambulatory 2^nd^ line care were assigned based on communication by the Dutch mental health association ‘GGZ Nederland’. For group trainings, the association estimated time used by the expert divided by the number of clients per session. For day-care, a weighted average of day-care treatment (8 h a day) and half-time treatment (4 h a day) was used (day-treatment €183.70 + half-time treatment €163.53). For parents, the average number of days of medication use in the entire group of parents per month was multiplied by mean public Dutch costs of medication use in adults (€0.66 per day) (Stichting Farmaceutische Kengetallen 2012). Direct non-medical costs related to weekend care and care after school were based on several sources as these were not defined in the costing manual. Weekend care was estimated based on productivity costs (Hakkaart-van Roijen et al. 2011). For remedial teaching, average price per hour for remedial teaching were used for cost calculation (Landelijke beroepsvereniging remedial teachers 2014). Care after school was rated based on average costs for baby-sitting (Nibud 2013). For diets, parents were asked to define costs below €25, between €25 and €50 or above €50 in one month if a special diet was indicated. For indirect non-medical costs, the agency for child welfare and police were assumed to have the same cost price since costs for police was not defined in the costing manual (Hakkaart-van Roijen et al. 2011). Prices for special education were based on prices of the Dutch Ministry of Education with a yearly additional cost of €4700 (Ministerie van Onderwijs 2012). This led to extra monthly costs for special primary and special secondary education of €392 compared to regular education. For the questions on child’s health-related school absence and failure to participate in sports due to ADHD no costs were assigned. Cost of absence from work of the parent related to their child with ADHD was calculated by multiplying the number of days off work with predefined productivity costs per hour of absence with a specification to age group and gender (Hakkaart-van Roijen et al. 2011). Costs of presenteeism was determined by the Osterhaus method (Osterhaus et al. 1992). Parents were asked about the number of days of hindrance due to ADHD of their child and their estimated efficiency on these days in comparison to usual efficiency. Linear extrapolation (LE) was applied as it was in a former ADHD study as well (Hakkaart-van Roijen et al. 2007). Applying LE, all costs were extrapolated to one year by multiplying the monthly costs by twelve assuming the monthly costs to reflect usual costs. There is no specific reason to assume that the cost distribution is non-linear, although there might be small variations during the year.

### Statistical analysis

Completeness of the data is reported based on numbers completing the questionnaire. Furthermore, the data was reviewed for fraudulent responses, errors (intentional or not), missing data and possible strange patterns indicating programming errors. Study variables are described using absolute numbers (mean and median), percentages and 95 % confidence intervals. Missing data was replaced by means of multiple imputation as is the recommended method to reflect estimates across the range of circumstances considered (Manca & Palmer 2005). Variables were considered candidates for the imputation process based on simple regression followed by a regression model based on significance and their independent influence on total costs. To check if distribution was normal, data were tested for skewness. Descriptive statistics, independent samples *t*-test and Chi-square test were used accordingly for analyzing differences between groups (p < 0.05; 2-tailed). As healthcare cost data tend to be skewed, tests were repeated using Mann–Whitney tests to check if conclusions remained similar using a test for non-parametric data. Multiple regression analysis was used to check for independent influence of other factors than treatment response on the mean total costs. A p-value of <0.05 was considered significant. It was not possible to examine test-retest reliability in this study, since the questionnaire was administered only once. Analyses were performed in IBM SPSS Statistics version 21.

## Results

### Demographic characteristics

Analyses were performed on 618 questionnaires of children and 590 questionnaires of parents (28 respondents – 4.5 % – did not complete all questions). Table 3 reports on the demographic characteristics for children and parents. ADHD had mainly been diagnosed by a psychiatrists or a pediatrician (67.8 and 17.8 %, respectively). Children were reported to be a responder to treatment in 428 cases (69.3 %) and non-responder to treatment in 190 cases (30.7 %). Mean age of children was 11.8 years. The number of comorbidities was similar between responder groups, although there was a near-significant difference of more children with three or more comorbidities in the non-responder group compared to the responder group (p = 0.051). Comorbidities in the entire sample were Asthma or chronic Bronchitis (n = 78; 13 %), Epilepsy (n = 7; 1 %), Anxiety disorder (n = 80; 13 %), mood disorder (n = 66; 11 %), learning disorder (n = 235; 38 %), Gilles de la Tourette/tic disorder (n = 25; 4 %), ODD/CD (n = 63; 10 %), mental retardation (n = 4; 0.6 %) and, Autism Spectrum Disorder (ASD) (n = 152; 25 %). Anxiety disorder (responder n = 48- 11.2 %, non-responder n = 32- 16.8 %; p = 0.049), and mood disorder (responder n = 38 – 8.9 %, non-responder n = 28 – 14.7 %; p = 0.023) differed significantly between the responder and non-responder group with more cases in the responder group.

**Table 3:** Children’s and parent’s characteristics

	Entire sample	Responder	Non-responder	p-value*
Children				
N	618 (100.0 %)	428 (69.3 %)	190 (30.7 %)	
Mean age	11.8 (8–18)	11.8 (8–18)	11.7 (8–18)	0.856
Gender				
Male	509 (82.4 %)	359 (83.9 %)	150 (78.9 %)	0.138
Female	109 (17.6 %)	69 (16.1 %)	40 (21.1 %)	
Number of comorbid disorders				
0	166 (27.6 %)	126 (30.0 %)	40 (22.1 %)	0.051
1	242 (40.3 %)	170 (40.5 %)	72 (39.8 %)	
2	132 (22.0 %)	89 (21.2 %)	43 (23.8 %)	
3 or more	61 (10.1 %)	35 (8.3 %)	26 (14.4 %)	
Type of education				
Primary	241 (40.2 %)	165 (39.3 %)	76 (42.2 %)	0.119
Special primary	86 (14.3 %)	57 (13.6 %)	29 (16.1 %)	
Secondary	219 (36.5 %)	165 (39.3 %)	54 (30.0 %)	
Special secondary	54 (9.0 %)	33 (7.9 %)	21 (11.7 %)	
Parents				
N	590** (100.0 %)	415 (70.3 %)	175 (29.7 %)	
Age (mean, range)	43.2 (30–63)	43.2 (30–63)	43.3 (30–59)	0.931
Gender				
Male	28 (4.7 %)	22 (5.3 %)	6 (3.4 %)	0.328
Female	562 (95.3 %)	393 (94.7 %)	169 (96.6 %)	
Marital status				
Together	534 (90.5 %)	381 (91.8 %)	153 (87.4 %)	0.097
Alone	56 (9.5 %)	34 (8.2 %)	22 (12.6 %)	
Education				
Lower	83 (14.1 %)	59 (14.2 %)	24 (13.7 %)	***
Medium	265 (44.9 %)	182 (43.9 %)	83 (47.4 %)	
Higher	232 (39.3 %)	166 (40.0 %)	66 (37.7 %)	
Other	10 (1.7 %)	8 (1.9 %)	2 (1.1 %)	
Paid job				
Yes	471 (79.7 %)	328 (79.0 %)	143 (81.3 %)	0.541
No	120 (20.3 %)	87 (21.0 %)	33 (18.8 %)	
More than 1 child with ADHD				
Yes	133 (22.5 %)	85 (20.5 %)	48 (27.4 %)	0.065
No	457 (77.5 %)	330 (79.5 %)	127 (72.6 %)	

*p value reflects differences between responders and non-responders

**28 parents did not complete the questionnaire

***Pearson Chi-Square not possible due to low expected numbers

Data on resource use for children was missing for 19 respondents and data on resource use and production losses for parents for 27 respondents. This missing data was replaced by means of multiple imputation. Data were imputed based on the most optimal regression model. For children, this model was based on responder group (responder vs. non-responder), gender of the parent, >1 child with ADHD in the family and comorbidity. For parents, data was imputed based on a model with responder group, >1 child with ADHD, comorbidity and marital status.

Table 4 presents the mean and median monthly costs and their 95 % confidence interval for direct medical and non-medical costs and indirect non-medical costs of children and parents. The group non-responding children incurred significantly higher total costs per month compared to responding children. This was reflected in total direct medical costs (€190 higher), direct non-medical costs (€74 higher) and indirect non-medical costs (€23 higher). Day-care, trainings and doctor’s consultation differed most remarkably between responder groups (€71, €65 and €57 higher for non-responders, respectively), with a significant difference for doctor’s consultations (1.3 visits responding children vs. 2 visits non-responding children; p = 0.004). Remedial teaching at school accounted for €33 extra per month for the non-responder group (€132 for responders, €165 for non-responders; p = 0.045). For the questions on child’s school absenteeism and failure to participate in sports no costs were assigned. Absence from sports due to ADHD was mentioned by the responder and the non-responder group (7.7 % vs. 16.8 % respectively, p = 0.002). School absenteeism was reported for 72 days by the responder group and for 50 days by the non-responder group (16.8 % vs. 26.3 % respectively, p = 0.003). Estimated annual costs for responders and non-responders to treatment were €3864 vs. €6144 for direct medical costs (p = 0.068), direct non-medical costs €2664 vs. €3552 (p = 0.090); indirect non-medical costs €408 vs. €684 (p < 0.001); and total costs for children €6936 vs. €10,068, p = 0.021 (responder vs. non-responder). Monthly costs per responder group are reported in Table 4.

**Table 4:** Mean (median; 95 % CI) costs of children with ADHD and parents per month (in Euro 2012)

	Entire sample	Responder	Non-responder	p-value*
Children				
Direct Medical costs				
Doctor’s consultations**	114 (30)	96 (0; 78 – 115)	153 (95; 123 – 193)	0.004
Trainings	126	106 (0; 69–148)	171 (0; 117 – 240)	0.068
Day-care treatment	73	51 (0; 11–84)	122 (0; 32 – 218)	0.144
Hospitalization	41	41 (0; −10 – 93)	41 (0; −37 – 125)	0.999
Medication	26 (12)	27 (12; 25–30)	24 (12; 20 – 29)	0.300
Diet	5	4 (0; 2 – 5)	6 (0; 4 – 10)	0.145
Total direct medical costs	386 (109)	326 (96; 222–422)	518 (145; 334–691)	0.068
Direct non-medical costs				
Weekend care	83	80 (0; 59 – 104)	91 (0; 60 – 122)	0.578
Remedial teaching at school	142 (110)	132 (0; 114 – 151)	165 (110; 137–194)	0.045
Care after-school	6	6 (0; 3 – 9)	7 (0; 2 – 12)	0.638
Total direct non-medical costs	232 (110)	218 (110; 191–253)	290 (150; 224–315)	0.090
Indirect non-medical costs				
Agency for child welfare	10	6 (0; 3 – 9)	20 (0; 9 – 20)	<0.001
Police	0	0 (0; 0 – 0)	1 (0; 0 – 1)	0.154
Special Education	30 (28)	28 (0; 23 – 33)	36 (0; 28 – 45)	0.090
Total indirect non-medical costs	41	34 (0; 28 – 40)	57 (0; 46–68)	<0.001
Total costs children	658 (322)	578 (266; 465–691)	839 (511; 648–1029)	0.021
Parents				
Direct Medical costs				
Doctor’s consultations*^,^**	60	47 (0; 39 – 57)	88 (33; 69–108)	<0.001
Trainings	93	81 (0; 54 – 109)	118 (0; 74 – 166)	0.145
Medication	1.60	0.93 (0; 0.5 – 1.3)	3.1 (0; 2 – 4)	<0.001
Total direct medical costs	154	130 (0; 98 – 162)	211 (82; 153–269)	0.010
Indirect non-medical costs				
Productivity losses due to absenteeism and presenteeism	136	116 (0; 74 – 159)	181 (0; 113 – 259)	0.092
Total costs parents	292 (65)	246 (21; 191–300)	399 (182; 298–482)	0.006
Children and parents together				
Total costs	948 (524)	824 (412; 690–957)	1228 (740; 1003–1453)	0.002

*p value reflects differences between responders and non-responders

**All consultations are summed up. Table 2 provides an overview specified per type of doctor

For parents, total monthly costs were significantly different between responder groups, incurring higher costs of €153 for a parent when having a child non-responding to treatment. Estimated annual costs were €2952 for the parents of responding children and €4788 for parents of non-responding children (*p* = 0.006). Both total direct medical and indirect non-medical monthly costs were higher for parents of a non-responding child with €81 (*p* = 0.010) and €65 (*p* = 0.092) respectively. As for children, doctor’s consultations differed largely between responder groups and this difference was significant (responder 0.75 per month vs. non-responder 1.43 per month; *p* = <0.001). Estimated annual costs were €1560 vs. €2532 for direct medical costs (*p* = 0.010); indirect non-medical costs were €1392 vs. €2172 (*p* = 0.092) and total costs were €2952 vs. €4788 (*p* = 0.006) (responder vs. non-responder).

Costs for children and parents combined led to a significant difference between responder groups in total costs with €404 extra per month when a child is a non-responder to treatment (monthly costs responder €824, non-responder €1228; p = 0.002). Estimated annual costs were €9888 for the responder group and €14,736 for the non-responder group (difference €4848; p = 0.002). Multiple regression showed that the factors treatment response, gender of the parent, >1 child with ADHD and comorbidity had an independent influence on total costs for children. For total costs of parents, the factors treatment response, >1 child with ADHD, comorbidity and marital status of the parent were independent influencers.

## Discussion

The purpose of this study was to estimate ADHD related costs for children and their parents taking a societal perspective associated with treatment response. Non-response to treatment was shown to be associated with significant higher direct and indirect costs both inside and outside healthcare for children as well as their parents. Estimated annual costs differed between response groups, with extra costs of €4848 for non-responders. Costs for special education were very low relative to the other cost items in this study. This could reflect little need for extra attention, but it can also be a reflection of the general national Dutch trend towards less use of special education (Dekker 2015). A study on the economic impact of ADHD in Europe concluded that the largest cost category was in fact in education representing 50 % of national costs on average (Le et al. 2013). The study did not include non-paid work based on the assumption that parents of children aged 8–18 would have limited voluntary activities. They could be coach of sports teams, be of help in their church or follow higher education. These costs were not included and could have impacted the absolute results. Most children were boys, which is consistent with other studies in ADHD. In the entire sample, only 28 % had no comorbidity and 10 % even had more than three comorbidities. This is consistent with the estimation that in general around 60–100 % of patients with ADHD exhibit one or more comorbid disorders (Faber et al. 2010; Gillberg et al. 2004). The percentage of 10 % of children with comorbid ODD/CD and 25 % of ASD in our study group seems to be low compared to other studies (Gillberg et al. 2004; Erskine et al. 2014). Anxiety disorder and mood disorder differed significantly between the responder and non-responder group with more cases in the responder group. This outcome was not expected, since comorbidity is expected to negatively influence ADHD outcomes (Gillberg et al. 2004).

Recently, a systematic review was performed on European studies of ADHD-related costs published between 1990 and 2013 (Le et al. 2013). Only seven studies were found. The studies were conducted in Belgium, the Netherlands, Germany (n = 3), Sweden and the United Kingdom. All studies included direct medical costs for children, but only one study included direct medical costs for family members whereas only two included costs of productivity loss of family members (indirect non-medical costs) (Hakkaart-van Roijen et al. 2007; Myren et al. 2010). Considering the methodology and the country of origin, only one study in the review can be compared well to the study at hand (Hakkaart-van Roijen et al. 2007). In that study, three groups were studied based on proxy reporting by the mother: 70 children treated by a pediatrician for ADHD, a non-matched group of 35 children with behavior problems and 60 children without behavior problems. The study showed the yearly direct medical costs of ADHD patients to be €2040. Moreover, ADHD children incurred yearly direct medical costs of €5908 which is in the range we found in our study (€3864 for responders to €6144 for non-responders). Indirect costs for the mothers of children with ADHD were €2243 for mothers compared to €1560 in our responder group and €2172 in our non-responder group.

It has been described before that studying ADHD is a challenge and concessions must be made considering time, money and information needed (Arnold et al. 1997). Limitations of our study are the following. First of all, a practical choice had to be made regarding selection of participants. As an alternative, the possibility to include parents of children already participating in a trial related to ADHD was discussed. It was decided not to do so, because of burden for participants and potential bias. We chose for parents who are a member of an ADHD parent association. Because of this membership, it was assumed valid to rely on the parents’ knowledge about the ADHD diagnosis of their child. Due to anonymity it was not possible to verify whether the diagnosis was valid or to collect data on disease severity. Similar considerations were stated in an earlier study, which used the same method of parent selection based on considerations of practicability, convenience and reliability (De Ridder & De Graeve 2006). With respect to generalizability of this data, future studies should take the specific study group in this study into account. Second, other than the descriptions provided by parents, no insight in actual medication use by means of for example a saliva test was available. This has been shown to provide different results compared to parent report (Pappadopulos et al. 2009). Third, the definition of non-responder in this study differs from clinical trials. These trials mainly aim at short-term effects and therefore at symptomatic improvement. This study aimed to focus on functional improvement as well. We explicitly broadened our definition of response and non-response, given our attempt to add to data generation on burden of illness in ADHD. Furthermore, it is questioned if current clinical trial designs in ADHD and response definitions are useful for measurement of individual treatment response anyway (Hodgkins et al. 2013). The definition we used provided a hands-on description of the study groups which provided easy reference to the respondents. Since we also wanted to ask questions on quality of life and costs for both the children and the parents we decided not to increase the questionnaire any further with a more in-depth description of functioning consisting of multiple questions. The statement of responders has been carefully assessed by the experts, but not by parents. We assumed the definition to be clear to parents, although the potential for alternative interpretations cannot be fully ruled out. Fourth, since we relied on a membership pool of an ADHD parent association, it could be the case that more severe ADHD cases were included in this study with a potential upward bias in the estimation of costs. Although, this is in conflict with the low percentage of comorbid ODD and ASD. Fifth, due to the cross-sectional methodology we cannot speculate about the causal relationship between these results and/or potential long-term benefits or disadvantages. A sixth point of interest in this study is that the definition of ‘response to treatment’ and ‘non-response to treatment’ includes some statement on impaired functioning of the non-responding child. One might postulate that impaired functioning a priori leads to higher costs. On the other hand, the response definitions did not include any statement on health care resource use which is the main focus of this study. Considering the remarkable difference in costs between both groups, the main conclusion of our study implying that non-response to treatment leads to higher costs seems to hold. We therefore assume the influence of this response definition to be modest only. Seventh, in case parents reported to have more than one child with ADHD, they were asked to report on their youngest child in order to reach consistency. The influence of this guidance is unknown. It could have resulted in underestimation of costs due to experience of the parent with the older child with ADHD, but also in overestimation since the youngest child was diagnosed most recently which could be associated with higher costs or younger children requiring more attention in general. Finally, next to pharmacological treatment, other types of treatment could have had an influence on the results since multimodal interventions are recommended for ADHD treatment (Daley et al. 2014; Trimbos-instituut et al. 2005). Pharmacological treatment together with interventions using behavioral techniques form the foundation for ADHD treatment (Trimbos-instituut et al. 2005). A meta-analysis of randomized controlled trials concluded that behavioral interventions have positive effects on a range of outcomes when used with patients with ADHD (Daley et al. 2014). Due to the method of our study it cannot be distinguished what the specific influence of either pharmacological treatment or behavioral interventions has been.

On the basis of our study, we would recommend future studies to use a random sample from multiple countries with possibilities to confirm diagnosis including validated checks on compliance, including validated questionnaires reporting on symptomatology (e.g. ADHD-RS-IV ADHD Rating Scale version 4 (Makransky & Bilenberg 2014)) and functioning (e.g. CGI-I Clinical Global Impression scale) (Busner & Targum 2007)), and existence of a control group using a longitudinal methodology with patient self-report, and parent and teacher report.

## Conclusions

The results of this study provide relevant information for health care provision and use, since the results stress the importance of focusing on achieving response to treatment in children receiving (pharmacological) treatment for ADHD. Direct medical costs for children - specifically day-care treatment and trainings - were identified to be the most disparate with higher costs in the non-responder group. Direct non-medical costs were also unequal between groups with the highest difference in coaching at school. For parents of children with ADHD, doctor’s consultations were higher in the responder group. Treatment pathways should incorporate a focus on achieving response to treatment reflected in recovery both at home and at school and proper compliance to prescription. Also family involvement should be a standard part of the pathway.

The results of this study contribute to the large evidence gap in societal cost calculation related to ADHD and emphasize the need for a broad perspective when analyzing problems related to ADHD since non-medical costs have a large contribution to total ADHD-related costs. With the trend of cost-effectiveness studies being used increasingly in guideline development and clinical decision-making, there is a clear need for comprehensive cost studies like the study at hand (Marsden & Wonderling 2013). As was described by others, programs to facilitate collaboration among payers, patients, employers, and educational institutions may provide opportunities to create strategies to consider the societal impact of ADHD and strategies to mitigate its impact (Doshi et al. 2012). This study may aid in indicating which parts of healthcare consumption and non-direct and indirect costs such strategy should primarily be focused on.

## Abbreviations

ADHD: Attention deficit hyperactivity disorder
ASD: Autism spectrum disorder
C: Compliance
CD: Conduct disorder
Ch: Children
F: Functioning
LE: Linear extrapolation
ODD: Oppositional defiant disorder
P: Parents
RIAGG: Dutch mental health facility: a combination of 1st and 2nd line care
TiC-P: Questionnaire on healthcare consumption and productivity losses for patients with psychiatric disorders
QoL: Quality of life
